# Esophageal schwannomas: A rarity beneath benign esophageal tumors a case report

**DOI:** 10.1016/j.ijscr.2019.03.038

**Published:** 2019-04-18

**Authors:** Emilio Sanchez-Garcia Ramos, Rubén Cortes, Alexandra Rueda de Leon, Emmanuel Contreras-Jimenez, Jorge Humberto Rodríguez-Quintero, Jesús Morales-Maza, Jorge Aguilar-Frasco, Alejandro Irigoyen, Frida Reyes, Alejandro Alfaro-Goldaracena

**Affiliations:** Oncology Surgery Department, Instituto Nacional de Ciencias Medicas y Nutricion “Salvador Zubiran”, Mexico City, Mexico

**Keywords:** Schwannoma, S100, Smooth muscle cell markers, Spindle-shaped

## Abstract

**Introduction:**

Esophageal Schwannoma is a rare tumor that represents the least frequent mesenchymal tumor of the esophagus and represents a condition with only a few cases reported in the literature

**Presentation of a case:**

We report a 40-year-old female with a 5 years history of gastroesophageal reflux, repeated history of pharyngitis, odynophagia that culminated in progressive oropharyngeal dysphagia to solids. A barium esophagogram revealed a filling defect in the superior and middle thirds of the esophagus. Upper gastrointestinal endoscopy showed a smooth elevated lesion in the upper third of the esophagus, impossible to resect by this mean. An open left cervical approach revealed an 80 × 45 × 20 mm large tumor, which was resected.

**Discussion:**

In general, Schwanommas are rarely found in the gastrointestinal tract (GI), while the great majority occur in the stomach, esophagic is the least common GI form of presentation.

**Conclusion:**

The knowledge about a new case, impacts in obtaining more information about the clinical course and surgical treatment of this tumor.

## Highlights

• Esophageal Schwannoma is a rare tumor with only few cases reported in the literature.
• In general, Schwanommas are rarely found in the gastrointestinal tract.
• Esophagic schwanommas is the least common gastrointestinal form of presentation.
• The knowledge about a new case of esophagic schwanomma, impacts in obtaining more information about the clinical course and surgical treatment of this tumor.

## 1 Introduction

Esophageal tumors are primarily malignant. Approximately 2% of all esophageal tumors are benign primary tumors [1]. Leiomyoma represents 80% of them, while esophageal Schwannoma is the least frequent mesenchymal tumor, and represents a condition with only a few cases reported in the literature [2]. In general, Schwanommas are rarely found in the gastrointestinal tract (GI), while the great majority occur in the stomach, esophagic is the least common GI form of presentation [3]. Diagnosis of these tumors is a challenge for the surgeon. The symptoms are vague and mostly asymptomatic, in part explained because of the slow growth rate. The most common symptoms, if present, are dysphagia and chest discomfort [4]. The present paper describes the case of a patient with a 5-year history of progressive oropharyngeal dysphagia in whom an esophageal submucosal tumor was resected. Diagnosis of esophageal schwannoma was confirmed by histopathological and immunohistochemical studies. A discussion on some of the related medical literature on this unusual subject is also presented. This work has been reported in accordance with the SCARE criteria [5].

## 2 Presentation of case

A 40-year-old female was admitted to our hospital with a 5 years history of gastroesophageal reflux, repeated history of pharyngitis, odynophagia that culminated in progressive oropharyngeal dysphagia to solids. Her medical and family histories were unremarkable.

**Fig. 1:**
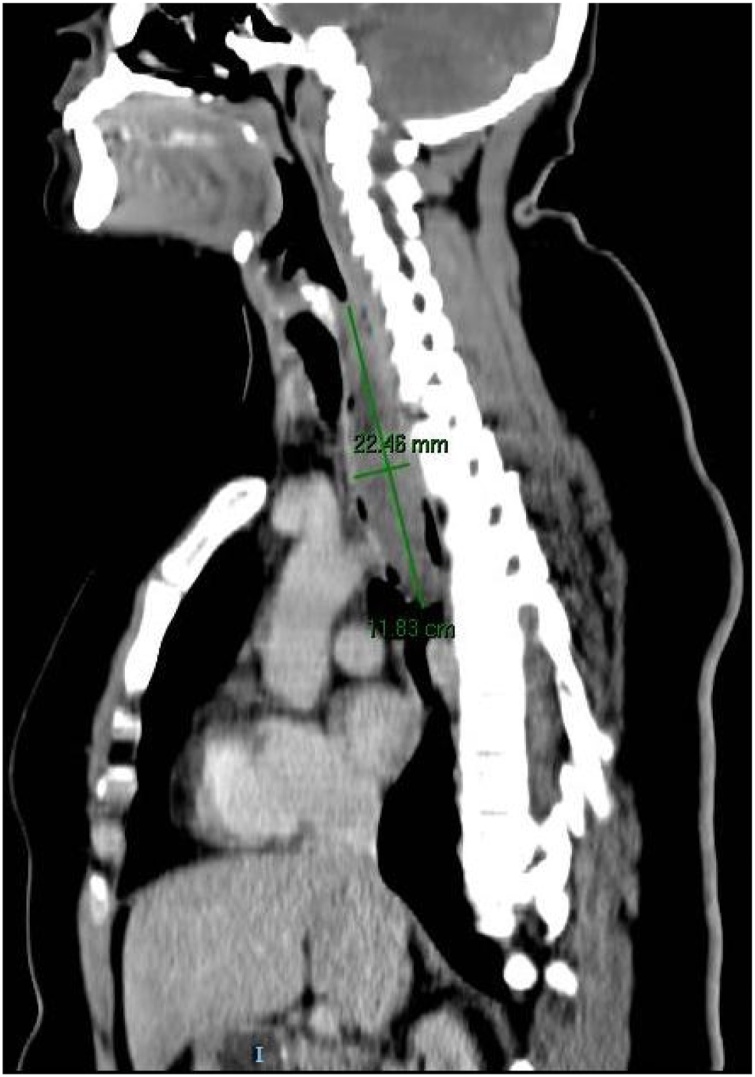
Computed tomography scan image showing a multilobulated esophageal tumor.

**Fig. 2:**
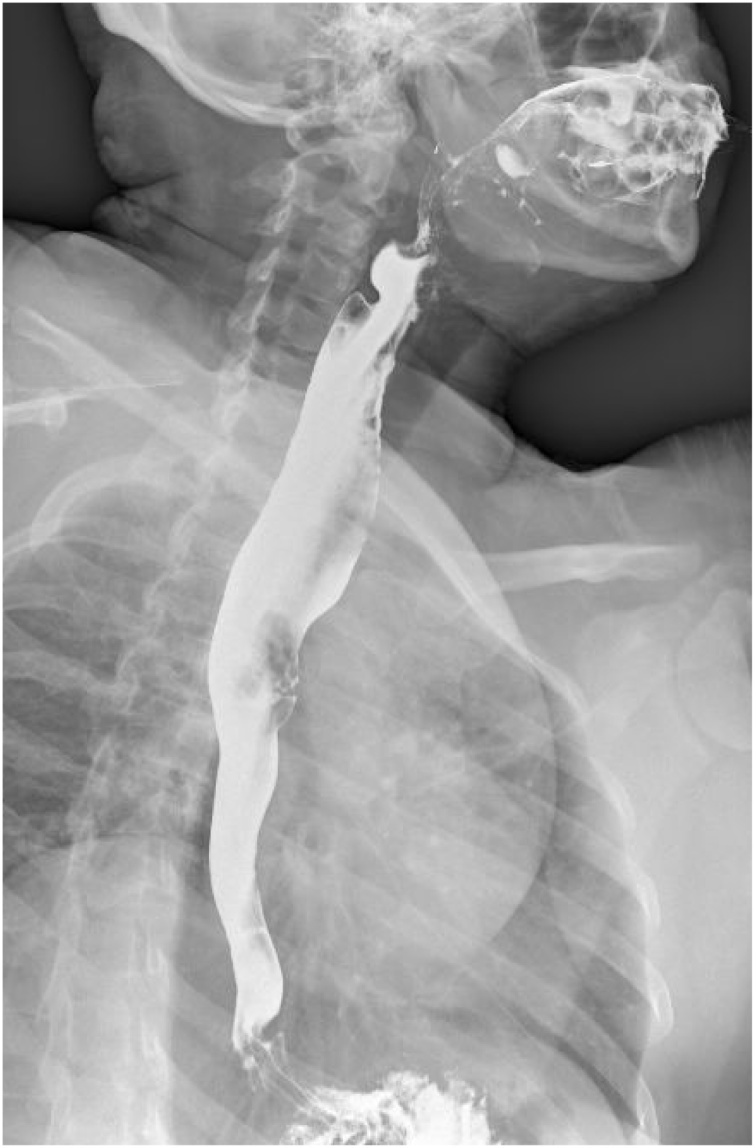
Barium esophagogram showing dilatation associated with a large protrusive tumor in the superior third of the esophagus.

A barium esophagogram revealed a filling defect in the superior and middle thirds of the esophagus (Fig. 1). A computed tomographic scan of the chest revealed a multilobulated low attenuation mediastinal mass of 32 × 20 × 12 mm (Fig. 2). We performed an upper gastrointestinal endoscopy which showed a smooth elevated lesion in the upper third of the esophagus which was impossible to resect by this mean.

**Fig. 3:**
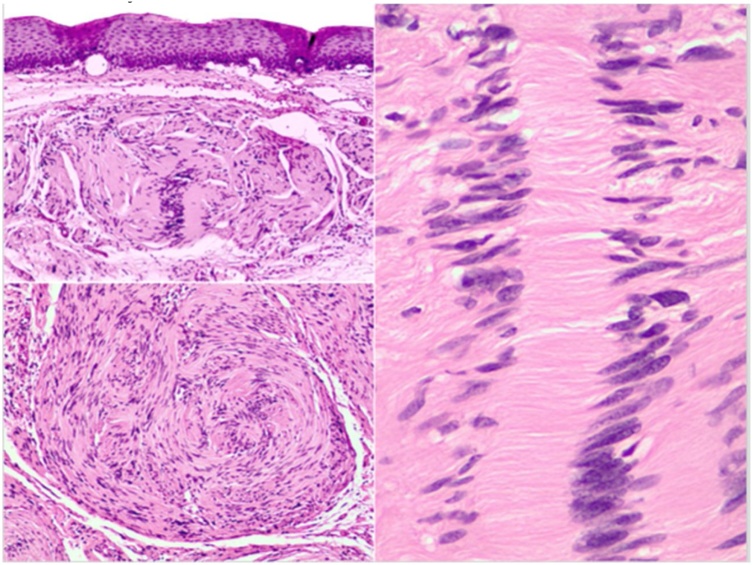
This histological image shows spindle-shaped bundles organized in a palisading pattern with anucleated areas composed of fibrillar material in parallel deposition corresponding to Verocay bodies.

Histopathological examination revealed a benign mesenchymal neoplasm formed by spindle-shaped bundles organized in a palisading pattern with anucleated areas composed of fibrillar material in parallel deposition corresponding to Verocay bodies (Fig. 3). There was an absence of mitotic activity and necrosis. Immunohistochemical studies revealed S100 protein positivity absence of staining for α smooth muscle actin (αSMA), CD34 and CD117, establishing the diagnosis of benign esophageal schwannoma.

**Fig. 4:**
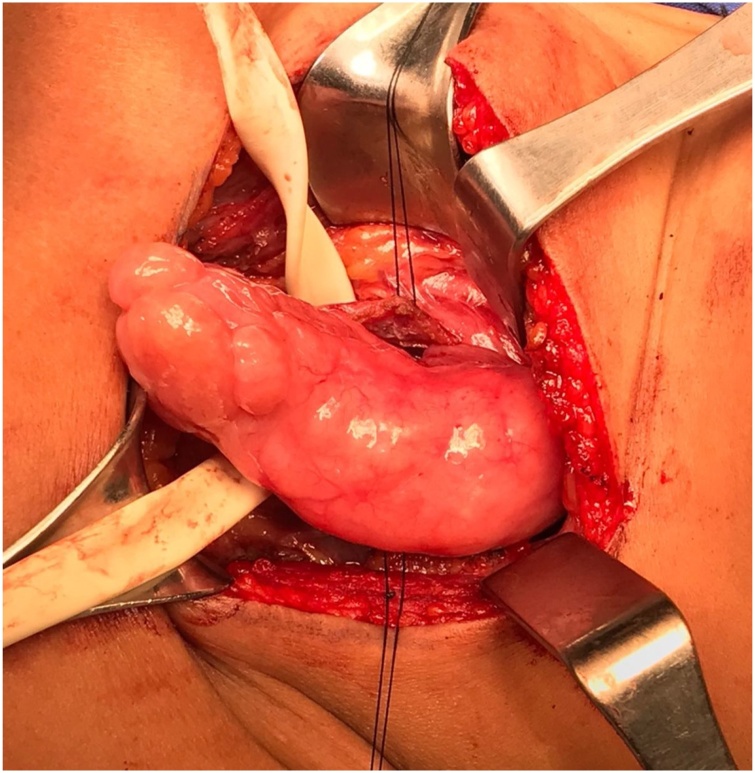
Lateral esophagus wall, exposing the esophagic tumor within the lumen.

**Fig. 5:**
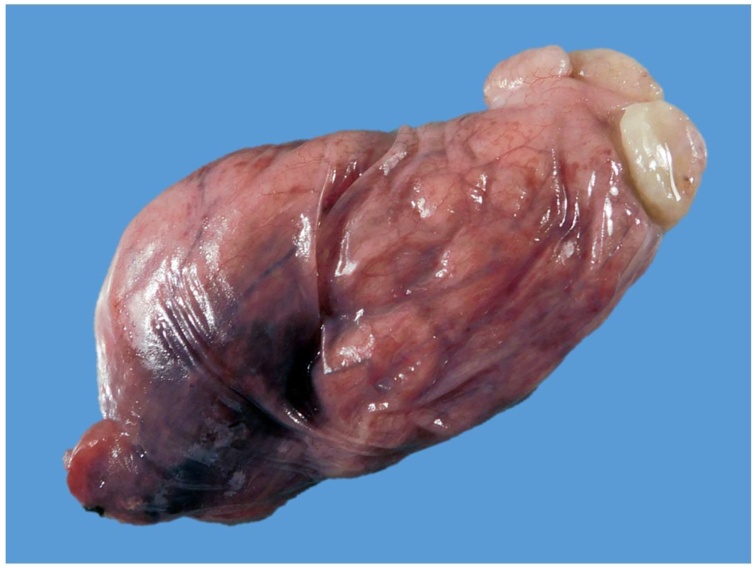
A macroscopic pathological image that shows a large (80 × 45 × 20 mm) smooth submucosal benign esophageal schwannoma.

This symptomatic mass was decided to be resected with an open left cervical approach. A “J” incision was performed to access to the visceral compartment of the neck until complete exposure of the cervical esophagus was achieved. A 5-cm longitudinal incision was performed in the external lateral esophagus wall, exposing the esophagic tumor within the lumen. The tumor was then luxated and externalized (Fig. 4). An 80 × 45 × 20 mm large smooth pedicle-dependent towards the anterior esophagus wall tumor was resected (Fig. 5). Esophagus defect was closed with polyglactin 910 simple suture. Her postoperative course was uneventful and there has been no evidence of recurrence to date.

## 3 Discussion

Benign nerve cell tumors have been given a variety of names, like Schwannoma, Neurinoma, Neurofibroma, Neurilemmoma and spindle cell tumors. They are the most common type of neurogenic tumor, derived from proliferating Schwann cells. Their origin site is usually the mediastinum [6]. Typically they are slow growing, homogeneous and mostly benign tumors, which rarely occur in the gastrointestinal (GI) tract, representing about 2–6% of all mesenchymal tumors [7]. The most common site of all GI cases is the stomach, followed by the colon and rectum. However, their location in the esophagus is extremely rare [8]. Esophageal Schwannomas are located most frequently in the upper esophagus, especially the cervical and upper thoracic regions. Sizes vary range from 0.5 cm to 16 cm. Park et al. reported a giant benign Schwannoma which measured 15 × 15 × 4.5 cm and weighed 720 g performing a total Thoracic esophagectomy [9].

Ever since Chaterlin and Fissore described the first esophageal schwannoma case around 1967 only 30 cases had been described till 2010 [10] and 13 more cases in the last 8 years reported in the medical literature (Table 1). Of the cases described, there is a strong predominance for this disease entity in the Asian population, with 16 of 25 cases reported from Asian institutions [11].

**Table 1:** Benign esophageal shwannomas case reports searched in Pubmed database during the last 8 years.

Author	Year	Age	Sex	Location	Depth	Size	Symptoms	Management
Choo et al.	2011	22	M	Upper thoracic esophagus	Submucosa	80 × 60 × 30 mm	Cough, dyspnea and dysphagya	Enucleation
Liu Tieqin et al.	2013	62	F	NA	Submucosa	90 × 40 × 30 mm	Dysphagia and dyspnea	Partial esophagectomy and esophagogastrostomy
Kitada M et al.	2013	55	F	Upper to middle mediastinum	Submucosa	75 × 57 × 80 mm	Palpitations and dysphagia	Mini Thoracotomy
Jeon Hyu Woo et al.	2014	32	F	Upper thoracic esophagus	Submucosa	87 × 59 × 24 mm	Chest pain	Surgical enuclation
Jeon Hyu Woo et al.	2014	63	M	Upper thoracic esophagus	NA	Two lobes: 95 × 70 × 65 mm 88 × 50 × 55 mm	No symptoms	Thoracotomy
Gu et al.	2014	39	M	Upper mid	Submucosa	35 × 32 × 12 mm	Obstructive sensation	Surgical esophagectomy
Tomono et al.	2015	59	F	Middle thoracic esophagus	Submucosa	109 × 7.2 × 7.1 mm	Dysphagia, dyspnea, disturbed consciousness	Subtotal esophagectomy
Wang, et al.	2015	53	F	NA	NA	NA	NA	Surgical excision
Wang, et al	2015	52	F	NA	NA	NA	NA	Surgical excision
Zhang Q, et al.	2016	67	F	NA	NA	NA	Dysphagia, foreign body	Surgical excision
Watanabe, et al.	2016	39	F	Upper third	Submucosa	55 × 45 × 24 mm	Epigastric pain, difficulty swallowing	Surgical excision
Moro K, et al.	2017	66	M	Upper third	Submucosa	52 × 40 × 31 mm	Dysphagia	Surgical excision
Onodera Y, et al.	2017	47	F	Aortic Arch	Submucosa	60 mm	Dysphagia	Thoracoscopic + endoscopic excision
Current article	2018	40	F	Upper third	Submucosa	80 × 45 × 20 mm	Pharyngitis, odynophagia, dysphagia	Surgical excision

NA: Not available.

The most common presentation age is between 50 and 60 years, However, Giuseppe et al. reported the case of a plexiform esophageal schwannoma in a child of 11 years old with neurofibromatosis type 2. In addition, there is a mild female predominance, with a male to female ratios ranging from 1:1.6 and 1:2.8 [12].

Diagnosis is made on a histological and immunohistochemical basis. Histologically, esophageal schwannomas are typically characterized by palisading spindle cells, few or absent mitotic figures, and a peripheral cuff of lymphoid cells containing germinal centers with anucleated areas composed of fibrillar material in parallel deposition corresponding to Verocay bodies. Immunohistochemically, tumor cells stain positive for S100, a characteristic marker of Schwann cells. Markers such as CD117 and CD34 will be negative, differentiating it from gastrointestinal stromal tumors (GIST). Smooth muscle cell markers (αSMA), actin and desmin, will also be negative, differentiating it from leiomyomas. Schwannomas are generally treated via surgical resection, but endoscopic resection can be done in lesion less than 2 cm. The surgical approach depends on the location of the lesion within the esophagus, surgeons can use the left cervical approach or the innovative video-assisted thoracoscopy to perform enucleation of the tumor [12].

## 4 Conclusion

We reported a rare case of a benign esophageal schwannoma. The knowledge about a new case, impacts in obtaining more information about the clinical course and surgical treatment of this tumor. The biological behavior of the esophageal schwannoma is in most cases benign, and the appropriate technique surgery depends on the location of the lesion.

## Conflicts of interest

Authors have nothing to declare

## Sources of funding

Authors have nothing to declare

## Ethical approval

The ethical approval for the publication of this case was exempted by our institution because all of the data were collected from clinical records and imaging systems for routine perioperative planning

## Consent

Informed consent was obtained from the patient for publication of this case report and any accompanying images, the corresponding author has it if it is needed.

## Author contribution

Emilio Sanchez-Garcia Ramos, Alejandro Alfaro-Goldaracena, Rubén Cortes: Conceptualization, Methodology, Writing -original draft preparation, Investigation, Supervision. Alexandra Rueda de Leon, Emmanuel Contreras- Jimenez: Data curation, Writing- Original draft preparation. Jorge Humberto Rodríguez-Quintero, Jesús Morales- Maza, *J*orge Aguilar -Frasco Investigation, Writing- Reviewing and Editing. Alejandro Irigoyen, Frida Reyes Visulization

## Registration of research studies

This is not a ‘First in Man’ study and should not be registered.

## Guarantor

Emilio Sanchez-Garcia Ramos

## Provenance and peer review

Not commissioned, externally peer-reviewed.
